# Primary Age-Related Tauopathy in Human Subcortical Nuclei

**DOI:** 10.3389/fnins.2019.00529

**Published:** 2019-05-29

**Authors:** Keqing Zhu, Xin Wang, Bing Sun, Juanli Wu, Hui Lu, Xiaoling Zhang, Huazheng Liang, Dandan Zhang, Chong Liu

**Affiliations:** ^1^China Brain Bank and Department of Neurology in Second Affiliated Hospital, Key Laboratory of Medical Neurobiology of Zhejiang Province, and Department of Neurobiology, Zhejiang University School of Medicine, Hangzhou, China; ^2^Department of Pathology, Zhejiang University School of Medicine, Hangzhou, China; ^3^Brain Structure and Function, Neuroscience Research Australia, Randwick, NSW, Australia; ^4^Department of Neurology, Shanghai Fourth People’s Hospital, Tongji University, Shanghai, China

**Keywords:** primary age-related tauopathy, subcortical nuclei, Alzheimer’s disease, neurofibrillary tangle, brainstem, brain bank

## Abstract

The present study aimed to determine the spatial distribution patterns of hyperphosphorylated tau-immunoreactive cells in subcortical nuclei of post-mortem human brain with primary age-related tauopathy (PART). Subcortical tauopathy has important pathological and clinical implications. Expression of tau was examined in different subcortical regions of definite PART cases with a Braak neurofibrillary tangle stage >0 and ≤IV, and with a Thal phase 0 (no beta-amyloid present). Post-mortem brain tissue of PART was studied using immunohistochemistry and subsequent semi-quantitative assessment with Braak NFT stage -matched pre-Alzheimer’s disease (AD) and AD cases as a control. Expression of tau was frequently found in subcortical nuclei including the substantia nigra, inferior colliculus, locus coeruleus, medulla oblongata in the brainstem, the caudate, putamen, nucleus globus pallidus in the striatum, the hypothalamus, thalamus, subthalamus in the diencephalon, and the cervical spinal cord in both PART and AD, but not in the dentate nucleus of the cerebellum. A positive correlation was found between the Braak NFT stage and the tau distribution (qualitative)/tau density (quantitative) in PART and AD. Brainstem nuclei were commonly involved in early PART with NFT Braak stage I/II, there was no preference among the substantia nigra, inferior colliculus, locus caeruleus and medulla oblongata. The prevalence and severity of tau pathology in subcortical nuclei of PART and AD were positively correlated with NFT Braak stage, suggesting that these nuclei were increasingly involved as PART and AD progressed. Subcortical nuclei were likely the sites initially affected by aging associated tau pathology, especially the brainstem nuclei including the substantia nigra, inferior colliculus, locus caeruleus and medulla oblongata.

## Introduction

The stepwise progression of tau pathology in Alzheimer’s disease (AD) is reflected by NFT Braak stages and this pathology is generally assumed to begin from the *trans-*entorhinal region (Braak et al., 2006). However, it has been shown that some subcortical nuclei are involved early, even at NFT Braak stage I or 0 (Braak et al., 2011; Attems et al., 2012b; Stratmann et al., 2016). Specifically, it has been shown recently that tau pathology is frequently seen in locus caeruleus (LC), suggesting that AD-associated tau pathology may begin from LC rather than from the *trans-*entorhinal region (Braak and Del Tredici, 2011; Braak et al., 2011). It is noted that LC was recently considered as the pretangle stage b before Braak stage I (Braak et al., 2011; Braak and Del Tredici, 2015), whereas the subcortical nuclei are in general not considered in the basic scheme of Braak staging. In fact, a number of other subcortical nuclei were reported to be involved in AD, including the hypothalamus, thalamus, and the substantia nigra (SN) (Mattila et al., 2002; Arendt et al., 2015; Theofilas et al., 2015). The studies have shown that these nuclei were involved early in the progression of the disease and the lesions had important clinical consequences (Rüb et al., 2017). Further studies are thus needed to determine how early and consistently these nuclei are affected (Braak and Del Tredici, 2012).

Primary age-related tauopathy (PART) is characterized neuropathologically by the presence of AD-type neurofibrillary changes without, or with few Aβ plaques. Definite PART has recently been defined by the absence of Aβ plaques (Crary et al., 2014). Whether PART is a subtype of early AD, or an individual aging related change, is still controversial (Duyckaerts et al., 2015; Jellinger et al., 2015). Due to the absence of Aβ plaques, PART shows a pure tauopathy. This is helpful for seeking the starting point of NFT “seeding” and the NFT progression mechanism (Crary, 2016). Subcortical tauopathy has been described in AD in the medulla oblongata (MO), SN, LC, and some other subcortical nuclei (Arendt et al., 2015). Tauopathy was also reported in the aging brain (Wharton et al., 2016), but this phenomenon has not been comprehensively described in PART. To address this issue, we examined tau pathology in the subcortical nuclei of definite PART cases that met the pathological criteria, Braak NFT stage-matched pre-AD, and AD cases. We found that tauopathy was frequently observed in the subcortical nuclei of PART, pre-AD, and AD, including SN, colliculus inferior, LC, MO in the brainstem; the caudatum, putamen, globus pallidus (GP) in the striatum; the hypothalamus, thalamus, subthalamus in the diencephalon. The severity (distribution and density) of tau pathology in these subcortical nuclei was significantly correlated with Braak stages and tauopathy in nuclei of the brainstem, striatum and diencephalon has important pathological and clinical consequences (Theofilas et al., 2015).

## Materials and Methods

### Materials

Two independent groups, including 16 neuropathologically confirmed definite PART brains, 7 AD (Braak NFT stage ≥V and CERAD plaque density C) and 5 pre-AD (Braak NFT stage Ø/IV and CERAD plaque density C) brains were selected for this study. All cases were obtained from the China Brain Bank, Zhejiang University School of Medicine. Subcortical nuclei were checked in five major brain areas: SN, colliculus inferior/pons (CIP), locus caeruleus/pons (LCP), and MO in the brainstem; the caudatum, putamen, and globus pallidus in the basal gangalia; the hypothalamus, thalamus, subthalamus in the diencephalon; the cerebellum (dentate nuclei) and cervical spinal cord (SCC).

The study included 16 PART cases: their age was 78.5 ± 9.17 years in the range of 60–98 years; 12 were male and 4 were female; their average brain weight was 1240 ± 70.71 g. 7 neuropathologically confirmed definite Pre-AD cases: their age was 80.9 ± 6.67 years in the range of 69–90 years; 2 were male and 5 were female; their average brain weight was 1152 ± 73.05 g. 5 neuropathologically confirmed definite AD cases: their age was 86.8 ± 6.94 years in the range of 79–99 years; 3 were male and 2 were female; their average brain weight was 1235 ± 136.73 g.

### Immunohistochemistry

Immunohistochemistry was performed on formalin-fixed, paraffin-embedded tissue from all autopsy cases. Small blocks of brain were dissected at autopsy and fixed in 4% paraformaldehyde (PFA) in 0.1 M phosphate buffer (pH 7.4) for 2 days. Following cryoprotection in 15% sucrose in 0.01 M phosphate-buffered saline (PBS, pH 7.4), blocks were cut at 3 μm thickness using a microtome. Free floating sections were incubated with 3% H_2_O_2_ for 10 min to eliminate endogenous peroxidase activity in the tissue. Prior to immunostaining, sections underwent microwave antigen retrieval for 15 min in the citrate buffer (pH 6.0). After washing with PBS containing 0.3% Triton X-100 (Tx-PBS) for 30 min, sections were blocked with 10% normal goat serum, and then incubated with the primary antibody (anti-hyperphosphorylated-tau, AT8: Mouse monoclonal, 1:200, Thermo Fisher Scientific, Rockford, United States; anti-amyloid β protein: Mouse monoclonal, 1:200, Sigma-Aldrich, St. Louis, United States) for 24 h in a cold room. Following treatment with the appropriate secondary antibody (anti-mouse), labeling was detected using the avidin–biotinylated HRP complex (ABC) system (Vector Laboratories, Burlingame, CA). The peroxidase reaction was carried out using a developer solution containing 0.4 mg/ml DAB and 0.0006% hydrogen peroxide in TBS. For negative control, the primary antibodies were omitted and all other steps carried out as described above. After the staining procedures, sections were mounted onto gelatin coated slides and dehydrated before being coverslipped with the DPX mounting medium.

The severity of tau pathology was semi-quantitatively scored based on a four-point grading scale (−/0, none; +/1, mild; ++/2, moderate; +++/3, severe). Of note, all cases initially were checked using the routine protocol for the Braak stage system.

### Data Analysis

The hyperphosphorylated-tau immunochemistry results were analyzed using the semi-quantitative tau score index (severity) and the qualitative tau score index (distribution). The quantitative tau score index was evaluated using a four-point grading scale, and the qualitative tau score index is evaluated using a two-point grading scale (negative, 0; positive, 1). For inter-group comparison of means, the Kruskal-Wallis H test was used. Statistical comparison between variables was performed using the Mann-Whitney test.

## Results

We investigated both the prevalence and severity of subcortical tau pathology in PART brains and compared them with those of pre-AD and AD brains. The present study correlated tau pathology in the subcortical nuclei with their Braak stages.

### Tau Pathology in Subcortical Nuclei in PART (Table 1)

**Table 1:** Subcortical nuclei tau distribution in PART.

PART	Age	Sex		Striatum		Diencephalon			Brainstem				CD	SCC
			Ca	Pu	Pa	HT	T	ST	SN	CIP	LCP	MO		
I	60	M	−	−	−	−	−	−	−	−	−	−	−	+
	74	F	−	−	−	−	−	−	−	−	−	−	−	−
	76	M	−	−	−	−	−	−	+	+	+	−	−	+
	80	M	−	−	−	−	+	+	+	−	++	−	−	+
	84	M	−	−	−	−	−	−	−	−	−	+	−	−
	91	M	+	+	+	−	−	−	+	+	−	−	−	−
II	70	M	+	−	−	−	−	−	+	+	+	−	−	+
	83	M	+	−	−	−	+	−	−	+	−	+	−	N
	65	M	−	−	−	−	−	−	−	+	+	−	−	−
III	80	F	N	−	−	++	+	+	++	+	N	+	−	+
	81	F	−	−	−	+	+	−	+	+	+	+	−	+
	83	F	−	+	+	+	+	−	+	++	+	+	−	−
IV	74	M	−	+	+	+	+		+	+	+	+	−	−
	78	M	+	−	+	+	−	+	+	+	−	+	−	N
	79	M	−	+	+	++	−	−	−	+	+	−	−	N
	98	M	+	+	+	++	+	+	+	+	+	+	−	+

*NFT, neurofibrillary tangle; Ca, caudate; Pu, putamen; Pa, globus pallidus; HT, hypothalamus; Th, thalamus; ST, subthalamus; SN, substantia nigra; CIP, colliculus inferior/pons; LCP, locus caeruleus/pons; MO, medulla oblongata; CD, cerebellum/dentate nuclei; SCC, cervical spinal cord. −, None; +/1, mild; ++/2, moderate; +++/3, severe. N not examined.*

**FIGURE 1:**
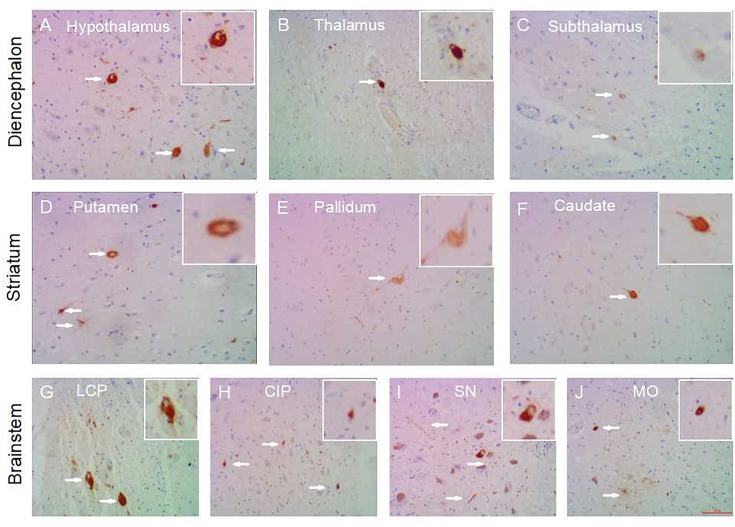
Subcortical nuclei tau distribution in PART. Tau pathology in the subcortical nuclei in PART cases: hypothalamus **(A)**, thalamus **(B)**, subthalamus **(C)** in the diencephalon; caudatum **(F)**, putamen **(D)**, globus pallidus **(E)** in the striatum; substantia nigra **(I)**, colliculus inferior/pons **(H)**, locus caeruleus/pons **(G)**, medulla oblongata **(J)** in the brainstem. All images are of the same magnification × 100. scale bars, 100 μm.

Among 16 PART cases, 15 cases (15/16) had tau-positive pathology in the subcortical regions. The region affected most frequently by tau pathology in the PART cohort was the brainstem including CIP (12 out of the 16 cases), SN (10/16), MO (9/16), and LCP (8/16); followed by the diencephalon including the thalamus (7/16), subthalamus (7/16), and hypothalamus (4/16); and the striatum including the caudatum (5/16), putamen (5/16), globus pallidus (6/16) (Figure 1). The SCC showed AT8 immunoreactivity (AT8-ir) in 7 out of the 16 cases (7/16). None of the cerebellum showed tau pathology (0/16).

The same regions were assessed using NFT Braak staging. In PART with Braak stage I, subcortical tauopathy was present in the brainstem (SN 3/6, CIP 2/6, LCP 2/6, MO 1/6), basal ganglia (caudatum 1/6, putamen 1/6, globus pallidus 1/6), thalamus 1/6, subthalamus 1/6 and SCC 3/6. PART cases with NFT Braak stage III/IV showed more AT8-ir in the brainstem (SN 6/7, CIP 7/7, LCP 5/7, MO 6/7), followed by the diencephalon (hypothalamus 7/7, thalamus 5/7, subthalamus 3/7), and the striatum (caudatum 2/7, putamen 4/7, globus pallidus 5/7). SCC showed AT8-ir in 3 out of 7 cases (3/7). Notably, the hypothalamus was consistently affected at Braak stage III /IV, but not at Braak stage I/II. None of the cerebellum showed tauopathy in PART cases.

In terms of tau distribution sites, the low grade (Braak stage I/II) PART showed AT8-ir in fewer subcortical sites (within 0–5 brain nuclei) than in the high grade (Braak stage III /IV) PART cases (between 5 and 11 nuclei). There was a statistical difference in the means of NFT distribution sites between PART with Braak NFT stage I/II and PART with Braak NFT stage Ø/IV (*p* < 0.01) (Figure 3A). The total tau IHC scores (four-point grading scale) were low in all subcortical nuclei of PART, below 1 at Braak stage I/II, and 1–2 at Braak stage III/IV. The tau score means of PART with Braak NFT stage III/IV cases were significantly higher than those of PART with Braak NFT stage I/II (*P* < 0.01) (Figure 3D).

### Tau Pathology in Subcortical Brain Sites in Pre-AD and AD (Table 2)

**Table 2:** Subcortical nuclei tau distribution in AD and pre-AD.

AD NFT (Braak)	Age	Sex	SP (CERAD)		Striatum		Diencephalon			Brainstem				CD	SCC
				Ca	Pu	Pa	HT	T	ST	SN	CIP	LCP	MO		
III	69	F	C	−	−	−	+	−	−	−	−	+	−	−	−
	79	F	c	+	+	+	+	+	+	−	++	+	−	−	−
	90	M	c	+	+	+	+	−	−	−	−	+	+	−	−
IV	78	F	c	−	−	+	−	−	−	−	++ +	++	−	−	−
	81	F	c	+	+	−	++	−	−	+	+	−	−	−	−
	83	M	c	+	+	+	+	+		+		+	+	−	−
	86	F	c	+	+	++	++	++	−	+	++	+	++	−	++
V	85	M	c	++	++	+++	++	++	++	++	+	+	–	−	+++
	99	F	c	+	+	+	+++	+	–	+	+	++	+	−	+
VI	79	M	c	++	++	++	+++	++	++	++	++	++	++	−	+
	86	F	c	++	++	+	+++	++	+++	++	++	++	++	−	N
	85	M	c	+	+	+	+	++	+	++	++	++	+	−	+

*AD, Braak NFT stage ≥IV and CERAD SP density = C; Pre-AD, Braak NFT stage III/IV and CERAD SP density = C or B. SP, Senile plaque; NFT, neurofibrillary tangle; Ca, caudate; Pu, putamen; Pa, globus pallidus; HT, hypothalamus; Th, thalamus; ST, subthalamus; SN, substantia nigra; CIP, colliculus inferior/pons; LCP, locus caeruleus/pons; MO, medulla oblongata; CD, cerebellum/dentate nuclei; SCC, cervical spinal cord. −, None; +/1, mild; ++/2, moderate; +++/3, severe. N not examined.*

**FIGURE 2:**
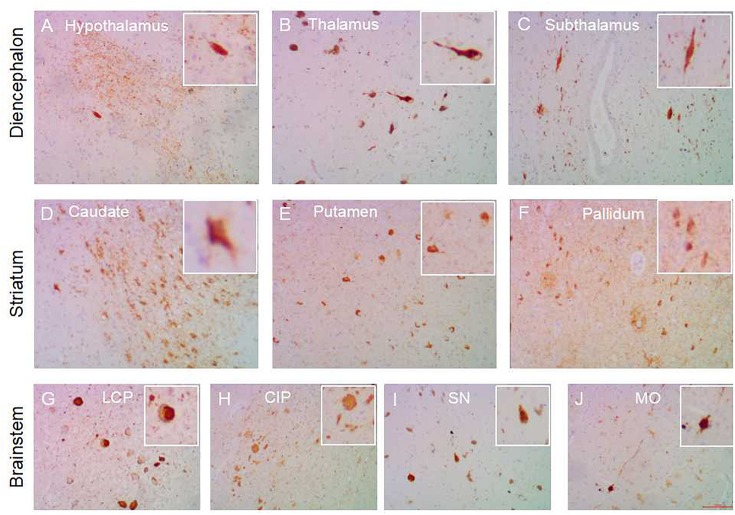
Subcortical nuclei tau distribution in AD. Tau pathology in the subcortical nuclei in AD cases: hypothalamus **(A)**, thalamus **(B)**, subthalamus **(C)** in the diencephalon; caudate **(D)**, putamen **(E)**, globus pallidum **(F)** in the striatum; substantia nigra **(I)**, colliculus inferia/pons **(H)**, locus ceruleus/pons **(G)**, medulla oblongata **(J)** in the brainstem. All images are the same magnification × 100. scale bars, 100 μm.

**FIGURE 3:**
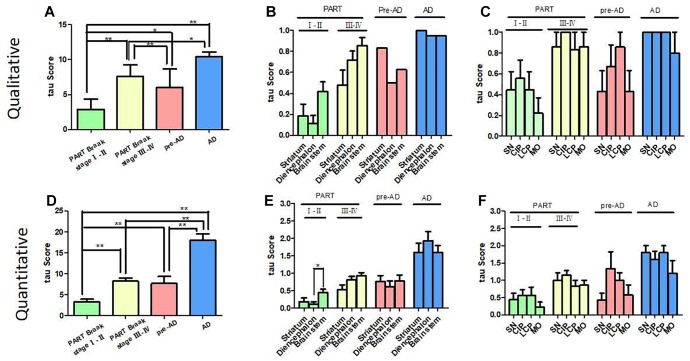
Correlation of Braak NFT stages with tau scores in PART and pre-AD/AD. **(A)** The qualitative tau score means (tau distribution pathology) in PART and AD/pre-AD. There was a statistical difference in NFT distribution score means between PART with Braak NFT stage I/II, PART with Braak NFT stage Ø/IV, pre-AD, and AD (*p* < 0.01). **(B)** The qualitative tau score means (tau distribution pathology) in the striatum, diencephalon, and brainstem in PART and AD/pre-AD. **(C)** The qualitative tau score means (tau distribution pathology) in SN, CIP, LCP, and MO in PART and AD/pre-AD. **(D)** The quantitative tau score means (tau density pathology) in PART and AD/pre-AD. There was a statistical difference in the tau score means between PART with Braak NFT stage I/II and PART with Braak NFT stage Ø/IV cases (*p* < 0.01) as well as pre-AD/AD (*p* < 0.01). **(E)** The quantitative tau score means (tau density pathology) in the striatum, diencephalon, and brainstem in PART and AD/pre-AD. **(F)** The quantitative tau score means (tau density pathology) in SN, CIP, LCP, and MO in PART and AD/pre-AD.

In all AD individuals, nearly all of the subcortical nuclei showed a marked to severe AT8-ir tau pathology (Figure 2). Compared with PART, the subcortical regions were frequently affected by tau pathology in pre-AD and AD cases. Tauopathy was frequently observed in the striatum (caudatum 10/12, putamen 10/12, globus pallidus 10/12), diencephalon (thalamus 8/12, subthalamus 5/12, hypothalamus 11/12), and brainstem (SN 8/12, CIP 9/12, LCP 11/12, MO 7/12). In pre-AD cases, fewer brain sites showed HP-tau immunoreactivity (i.e., within 2–8 sites) than in AD (10–11 sites). A significant difference was found in the number of sites affected between pre-AD and AD (*p* < 0.01), between PART with Braak NFT stage I/II and pre-AD/AD (*p* < 0.05), and between PART with Braak NFT stage III/IV and AD (*p* < 0.05). There was no significant difference in the number of sites affected between pre-AD and PART with Braak NFT stage III/IV (*P* > 0.05) (Figure 3A).

In terms of tau density, similar to late PART with Braak stage III/IV, Pre-AD cases showed minimal AT8-ir with a total tau score of 1–2 in subcortical nuclei, whereas nearly all AD cases showed a higher tau score of 2–3 in the majority of subcortical nuclei. A statistical difference in the means of tau scores was observed between pre-AD and AD (*p* < 0.01), between PART with Braak NFT stage I/II and pre-AD/AD (*p* < 0.01), between PART with Braak NFT stage III/IV and AD (*p* < 0.01). There was no statistical difference in the means of tau scores between pre-AD and PART with Braak NFT stage III/IV (Figure 3D). Similarly, total tau scores increased with the increasing NFT Braak stages in SCC, scoring 1 in pre-AD and 3 in AD. No AT8-ir was seen in the cerebellum of either AD or pre-AD cases.

The tauopathy distribution scores of the striatum, diencephalon and brainstem showed no significant difference between AD and pre-AD cases (*P* > 0.05). In PART, especially in the early Braak stage I/II, many brainstem nuclei showed tauopathy (Figure 3B) with a moderate to high severity score. Because of the limitated number of cases, a statistical difference was only found between the brainstem and the diencephalon in PART with Braak stage I/II (Figure 3E). There was no significant difference in the distribution (Figure 3C) and severity (Figure 3F) scores between brainstem nuclei including SN, CIP, LC, and MO.

## Discussion

Recent studies indicated that tau pathology in AD did not initially manifest in the cerebral cortex but in selected subcortical nuclei, including the thalamus, striatum and brainstem, in particular LC (Elobeid et al., 2012). Structural brain imaging studies also found changes in subcortical regions in early stage AD (Tentolouris-Piperas et al., 2017). For example, the thalamus and striatum were found to be atrophied in symptomatic patients, with an altered caudate volume implicated in early stage AD (Leh et al., 2016). Without Aβ deposition, PART represents a pure tau pathology at the early stage of neurodegeneration and is a good model of studying the mechanism for NFT Braak staging. In a previous study (Attems et al., 2012a), we addressed the question whether degeneration of subcortical nuclei occurred early during the progression of PART. In the present study, we systematically assessed the subcortical nuclei in PART and per-AD/AD patients ranging from preclinical stages to severe dementia. In the five brain regions we checked, tau became detectable in the brainstem, diencephalon, striatum, and spinal cord in PART and pre-AD, but not in the cerebellum. Tauopathy was more pronounced in these regions in more advanced AD with higher NFT Braak stages.

### Primary Age-Related Tauopathy in the Brainstem

It was reported that brainstem nuclei were affected by early AD before the supratentorial regions, including the LC, SN and the nucleus basalis of Meynert (NbM). Some normal aging subjects without NFTs (Braak 0) in LC showed NFTs in the dorsal raphe nucleus (DR) (Grinberg et al., 2011). In the present study, LC showed tau pathology in early PART with NFT Braak stage I, other brainstem nuclei including SN, CIP and MO also showed tau pathology at the same time. In some PART cases with NFT Braak stage I, a few brainstem nuclei, such as the MO (Table 1/ case 5), SN, and CIP (Table 1/case 6), but not LC, showed tau pathology. So at least, when LC was involved, some other brainstem nuclei have already manifested tauopathy in early PART with NFT Braak stage I. Compared with the striatum and the diencephalon, brainstem nuclei were the most commonly affected region at the early stage of PART with NFT Braak stage I/II, which supports that tauopathy may begin from the brainstem (Simic et al., 2009; Lee et al., 2015; Rüb et al., 2016b). In pre-AD and AD brains, tau pathology was severe and evenly distributed in these nuclei without a clear preference (Figure 3B,E).

### Primary Age-Related Tauopathy in Striatum and Diencephalon

In our study cohort, tauopathy was also positive in the other subcortical nuclei including the caudate nucleus, putamen, globus pallidus in the striatum, the thalamus and subthalamus in the diencephalon in early PART with NFT Braak stage I. Early tauopathy is not confined to a single subcortical nucleus. The possibility that neurodegeneration occurs independently at a number of sites in parallel cannot be ruled out (Rüb et al., 2016a). Moreover, more subcortical nuclei were positive for tau in PART with higher Braak stages like III/IV. Tau pathology (both distribution and density) in the diencephalon and striatum showed a nearly identical pattern as that shown in the brainstem. In the hypothalamus, thalamus, and subthalamus, tau pathology (density and distribution) was negative or mild in early PART (NFT Braak stage I/II), moderate in late PART (NFT Braak stage III/IV) and pre-AD (NFT Braak stage III/IV), and severe in AD brains. Nuclei in the striatum (caudatum, putamen, globus pallidus) showed a similar pattern. We propose, therefore, that the subcortical nuclei should be considered in the basic scheme of Braak NFT staging in the future as others suggested (Paskavitz et al., 1995; Mattila et al., 2002; Pievani et al., 2013; Kawakami et al., 2014).

### Primary Age-Related Tauopathy in the Spinal Cord and the Cerebellum

To date, there are few studies having examined deposition of abnormally phosphorylated tau in the spinal cord of normal aging subjects or AD patients. A study showed that the cervical cord segments were affected in 96% AD vs. in 27% non-demented individuals (Dugger et al., 2013). We found that SCC was frequently positive for tau pathology (11/12, 92% in AD vs. 7/16, 44% in PART) in both AD and PART brains, and was even positive in early PART with Braak stage I. No tauopathy was observed in the cerebellum/dentate nuclei in our PART and AD brains.

### Subcortical Tauopathy Has Important Clinical Implications

**FIGURE 4:**
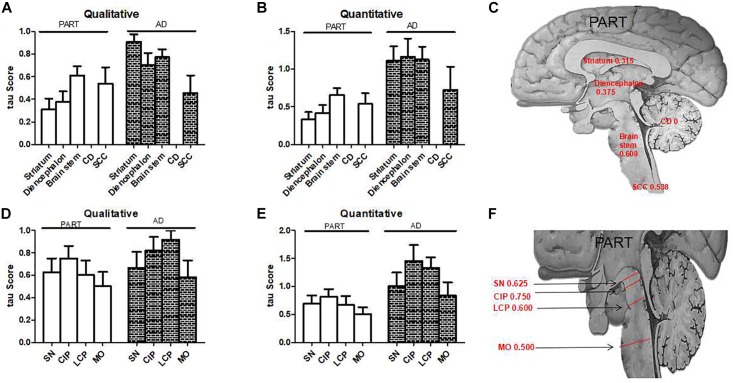
The qualitative and quantitative tau scores in PART and AD. **(A)** The qualitative tau score means in subcortical nuclei in PART and AD. **(B)** The quantitative tau score means in subcortical nuclei in PART and AD. **(C)** The qualitative tau score means in the striatum, diencephalon, brainstem, and cervical spinal cord in PART. **(D)** The qualitative tau score means in brainstem nuclei in PART and AD. **(E)** The quantitative tau score means in brainstem nuclei in PART and AD. **(F)**. The qualitative tau score means in SN, CIP, LCP, and MO in PART.

In the present study, tauopathy was observed in subcortical nuclei of PART brains. Based on the tau score (both qualitative and quantitative scores), brainstem was the most frequently affected region by tauopathy, followed by the diencephalon, striatum and SCC in PART (Figure 4A–C). Among these brainstem nuclei, there was no evident prevalence tendency observed (Figure 4D–F). The presence of tau pathology in subcortical nuclei has important implications for both the pathogenesis and clinical manifestations of PART and AD. As tau immunoreactivity is present in the subcortical regions of PART as well as pre-AD subjects, it could explain some clinical symptoms prior to typical dementia symptoms manifest (Besser et al., 2017; Josephs et al., 2017). The density (tau score) of AT8-ir cells increased in all regions investigated as the NFT Braak stage increased in AD, which could explain symptoms frequently found in AD. But this has not been correlated with tau pathology in the subcortical nuclei such as SN or LC in PART (Giaccone, 2015). Tau pathology in the brainstem is severe in AD, which may explain clinical symptoms due to serotonergic deficit found in AD, and a variety of less well-understood symptoms of AD patients. For example, parkinsonian extrapyramidal motor signs, depression, hallucinations, dysfunctions of the sleep/wake cycle, etc. (Attems et al., 2007).

Although there are some reports about the subcortical tauopathy in AD, the extent of the subcortical tauopathy in aging and AD has been underestimated (Janocko et al., 2012; Lemche, 2018). To our knowledge, this is the first systemic report on the occurrence of tau accumulation in the subcortical regions in PART. Our findings showed that tau pathology began from the subcortical nuclei in PART as early as NFT Braak stage I. The distribution and density of tau pathology in the subcortical nuclei significantly increased as the NFT Braak stage increased in both PART and AD. These observations indicate that subcortical nuclei are inflicted by neurofibrillary changes as early as the *trans-*entorhinal cortex in both PART and AD. Study of tau pathology in the subcortical nuclei improves our understanding about the evolution of clinical manifestations of AD and provides a simple and early structural indicator of PART and AD development. Prevalence of abnormal tau accumulation in the subcortical regions in PART and AD may support the hypothesis that abnormal tau aggregation propagates via neural circuits. PART will be an optimal disease model for testing hypotheses related to tau propagation in the brain.

## Ethics Statement

The research was given ethical approval by Medical Ethics Committee of Zhejiang University School of Medicine.

## Author Contributions

XW, BS, JW, XZ, and HL did the immunohistochemistry analysis and tissue preparation. KZ and XW contributed to statistical assessment and data processing. KZ designed the study, supervised the results, and wrote the first advanced version of the manuscript which was circulated among all the contributors for comments and suggestions. HL, DZ, and CL contributed to the final version of the manuscript.

## Conflict of Interest Statement

The authors declare that the research was conducted in the absence of any commercial or financial relationships that could be construed as a potential conflict of interest.
